# Why Not? A Plea

**Published:** 1907-01

**Authors:** James A. Gardner

**Affiliations:** Buffalo. N. Y.; Attending Surgeon to the Genito-Urinary Department, Emergency Hospital


### Why Not? A Plea.

By JAMES A. GARDNER, M. D., Buffalo, N. Y.
Attending Surgeon to the Genito-Urinary Department, Emergency Hospital.

THE most powerful aid to the progress of preventive medicine is the education of the public to an appreciation of the need and value of prophylaxis. The more extensive the medical knowledge of the laity, the greater are the benefits they receive from the medical profession. This has been markedly demonstrated in recent years in the care and prophylaxis of tuberculosis. Such being a fact, why is it that a department of medicine as important to the human race as that of venereal diseases is looked upon by the public with indifference, if not actual opposition to any enlightenment? The general public knows little or nothing respecting the dangers of these diseases.

The experience of all medical men shows that ignorance is responsible for a large proportion of infections in the young, and that enlightenment, which engenders a wholesome fear of these diseases, would preserve thousands from their contraction. Education in this direction would not only serve as a preservative against exposure to infection, but would constitute the most valuable safeguard against the introduction of such diseases among the married. The large majority of men who carry disease and death into their families from uncured venereal diseases do so through ignorance. A general diffusion of knowledge regarding the nature and danger of such diseases, the duration of their contagious activity and the terrible consequences to wives and children would be largely instrumental in preventing these social crimes.

Think of the many lives wrecked by venereal diseases! The The tabetics in our streets, the paretics in our asylums, the dead or disfigured children; think of the laparotomies for pus tubes that occur daily in our hospitals; the impotence, the sterility, the sexual neurosis that fill our offices with vagrants wandering from physician to quack and back again in the vain hope of cure, and then question the necessity of the education of the public as to the care and prophylaxis of venereal diseases! Does it not seem absurd?

Since the medical profession is the repository of this knowledge, it is evident that physicians must be its chief purveyors to the public. In dealing with a class of cases whch are contagious during a prolonged period, the prompt suppression of the sources of contagion by treatment is the most effective measure to prevent the spread of infection. The cure of the individual and the prevention of disease should go hand in hand. The physician must not only realize the serious responsibility of the case, but must be prepared by a theoretical and practical knowledge of these diseases to apply up to date methods and appliances of diagnosis and treatment.

How often do we find the gonorrheic patient received by the physician with a smile of levity, and his "little misfortune" made the subject of a joke. No wonder the popular mind considers that the treatment of venereal diseases requires no special medical knowledge or skill and that those infected do not hesitate to entrust their cases to the drug clerk, the advertising quack, or the merest novice in medicine; or that they even avail themselves of the prescription of an obliging friend, all with the deplorable result that the vast majority are improperly and insufficiently treated and not definitely cured, but go on scattering broadcast the seeds of dangerous infection which by proper treatment might have been sterilized.

I dare say there is no class of disease in any department of medicine, which in the past has been so neglected, mismanaged or which has received such a routine or unscientific treatment as the venereal class. Many physicians are derelict in the performance of their duty by declining to treat the patient; they do him an injury which is so far reaching as to affect the general public. The average patient knows so little of venereal diseases that the fact that the physician in whom he has confidence will not treat him, makes him apprehend that he has an ailment which is beneath a dignified consideration by the medical adviser. This inference readily leads to the conviction that he has a "shameful" disease. The patient is entitled to treatment, as much at least as the individual who acquires a disease in consequence of drunkenness or other infraction of morals or ethics.

Gonorrhea is still looked upon by many physicians as a trivial affection and their entire equipment consists of a glass syringe and half a dozen formulæ for injections. We chide the public for that ridiculous prudery which looks upon education in sexual hygiene as improper, as demoralizing even for the young, and for that traditional prejudice, which surrounds sexual disease with an atmosphere of shame. From the standpoint of science there can be no greater satire upon creative wisdom than the idea that the knowledge of the organs that transmit life is shameful, or that the education which would lead young men to live according to the physiologic laws of a healthy nature is profane.

We censure the public for its insensibility to the significance of the venereal peril, its indifference, its hostility, even to measures of prevention. Does not the public take its cue from the attitude of the sanitary officers who are charged with the health of the people and yet entirely ignore the existence of these diseases in the registration of those included as contagious? Are not these diseases excluded from our hospitals when they are acute and curable, and most dangerous as a source of contagion to others? If they are admitted they must be disguised under some evasive term, so that reliable statistics are impossible to compile. And yet it is generally admitted by recent writers that gonorrhea is the most widespread and universal of all diseases. Different writers claim that from seventy-five to ninety percent of the male population of our cities have had gonorrhea some time during their lives. Noeggerath has stated that of every one thousand men married in New York, eight hundred have had gonorrhea from whom the great majority of their wives have also been infected.

Morrow states that his own observations at the New York Hospital extending over a period of several years, would indicate that fully seventy percent of all women who had come there for treatment were respectable married women who had been infected by their husbands. Lydston may be most appropriately quoted on this subject. He says:

Why the profession should join the ignorant in tabooing sexual knowledge is a mystery, quite as much so as a great deal of other cant and hypocrisy that has pervaded the medical profession from time immemorial. So-called ethics has done more to foster quackery than to prevent it. And the public smiles derisively at a profession which after years of travail, will tolerate "practice limited to diseases of women" on professional cards; yet would roll up its eyes like a dying rabbit should it perchance run across a card inscribed: "Diseases of men only." Precisely what phase of sentiment elevates a woman with leucorrhea to a higher plane than that which a man with spermatorrhea occupies is one of the things which, as Dundreary says, "no fellow can find out." Irrespective of cause, it is a deplorable fact that the regular profession is woefully ignorant and culpably negligent regarding the sexual ailments of its clientele. If the public is to be educated the responsibility lies with the profession. The sexual organs are the noblest attributes of man, and their diseases are quite as worthy of intelligent study and considerate treatment as the affections of other organs. False modesty and mawkish sentiments have no place in medicine.

Since prostitution is the fountainhead of this disease, it might seem that the only effective remedy would be to attack the evil at its source. All experience proves, however, that prostitution must be looked upon as a necessary evil in our social system which cannot be uprooted or destroyed, and it must be remembered that it is not the prostitute, but the husband and father, who carries the poison home and distributes it to his family.

The true remedy, the only remedy at the present available to modify or minimize the appalling evils, moral and physical, which flow from venereal diseases, is the education of the public, the general dissemination of knowledge respecting the dangers and modes of contagion of venereal diseases. It is not by legislative enactments, but by the persuasive force of enlightenment, by combating the dense ignorance which prevails among the laity, and especially among the young upon whom the incidence of these diseases most heavily falls that these evils can be corrected.

If a young man is instructed in the knowledge of the facts that venereal disease is the almost invariable penalty of licentious living, that such indulgence is not wholesome for him, that it carries with it consequences to himself and others, consequences which may impair his health, vitiate his manhood and lead to a forfeiture of all those hopes and aspirations which are to be fulfilled in a safe, fruitful and happy marriage,—he will pause and consider.

What the laity needs then is such enlightenment. It is through the medical profession that this saving and salutary influence of knowledge must come. The family physician is peculiarly adapted, by his intimate relation with his patients, the freedom which his vocation allows him to talk on topics ordinarily forbidden, and his relation as friend as well as professional adviser, to impart this information and explain matters relating to sexual hygiene in a manner always decent but sufficiently plain.

403 Franklin Street.

---

Earache.—Albert Bardes emphasizes the fact that the importance of this symptom is still not recognised as it should be by either the laity or the profession, and he discusses the nature, etiology, and symptomatology of middle ear infections. Of the treatment he says that as soon as earache begins the patient should be kept quiet, put to bed and placed upon a fluid diet, and in other ways treated as we would treat a patient with a high fever. The bowels are to be kept open, and a single dose of morphia may be given to insure rest and comfort. Dry heat or else an ice-bag can be applied to the ear. The former is more acceptable to most patients. Every three hours the ear should be gently irrigated with a hot solution of bichloride 1 to 5,000, after which a few drops of a 12 per cent. solution of carbolglycerine can be instilled. Under no consideration should a person be allowed to suffer pain longer than twenty-four hours. If the pain continues, and the drumhead is inflamed and distended, pallative measures are worse than useless, and any attempt to abort the inflammation by means other than surgical is dangerous, and valuable time is lost in so doing. A bulging drumhead should be treated in the same way as a septic formation in any other place. It should be freely incised, rather than simply punctured or allowed to break.—*Medical Record,* January 20, 1906.

